# Transfer learning for T-cell response prediction

**DOI:** 10.1186/s12859-026-06492-2

**Published:** 2026-06-10

**Authors:** Josua Stadelmaier, Brandon Malone, Ralf Eggeling

**Affiliations:** 1 Department of Computer Science, University of Tübingen, 72076 Tübingen, Germany; 2 NEC OncoImmunity, Oslo, Norway; 3 Quantitative Biology Center (QBiC), University of Tübingen, 72076 Tübingen, Germany; 4 Institute for Bioinformatics and Medical Informatics (IBMI), University of Tübingen, Tübingen, Germany

**Keywords:** T cells, MHC, Peptides, Shortcut learning, Domain adaptation, Transformers

## Abstract

We study the prediction of T-cell response for specific given peptides, which could, among other applications, be a crucial step towards the development of personalized cancer vaccines. It is a challenging task due to limited, heterogeneous training data featuring a multi-domain structure; such data entail the danger of shortcut learning, where models learn general characteristics of peptide sources, such as the source organism, rather than specific peptide characteristics associated with T-cell response. Using a transformer model for T-cell response prediction, we show that the danger of inflated predictive performance is not merely theoretical but occurs in practice. Consequently, we propose a domain-aware evaluation scheme. We then study different transfer learning techniques to deal with the multi-domain structure and shortcut learning. We demonstrate a per-source fine tuning approach to be effective across a wide range of peptide sources and further show that our final model is competitive with existing state-of-the-art approaches for predicting T-cell responses for human peptides.

## Introduction

The human immune system consists of humoral and cell-mediated mechanisms, with T cells playing a significant role in the latter. T cells recognize and eliminate cancerous or infected cells by detecting peptides presented on the cell surface by major histocompatibility complex (MHC) molecules, which can be grouped into two classes, MHC I and MHC II [1].

Peptide-based vaccines are a promising approach for the personalized treatment of cancer [2] and might allow more precise vaccines against infectious diseases [3]. Such vaccines can be developed by selecting peptides that are specific to a pathogen or tumor. Since only a limited number of peptides can be included in the vaccine, their selection should be based on their probability of inducing a T-cell response. Predicting this probability is an important task in the development of peptide vaccines [2].

From a computational perspective, this task can be separated into two sub-tasks: first predicting whether a peptide is presented on the cell surface by an MHC molecule and afterwards predicting whether a presented peptide induces a T-cell response. For the first task, a large amount of available experimental data has led to accurate machine learning-based predictions [4–6]. The second task can, despite some attempts [7, 8], still be considered as an open problem, in part due to fewer experimental data from T-cell assays [9].

Aside from small sample sizes, another challenge arises from peptides in T-cell response data originating from different sources, such as viruses, bacteria, or human proteins. Moreover, peptides feature patterns that are specific to the allele of the MHC molecule they are presented by [10]. Both factors lead to data heterogeneity, which is either unaccounted for by existing approaches [8, 11] or handled by limiting the model to selected MHC alleles and considering small peptides, such that positions that are affected by MHC allele-specific patterns can be identified [7, 12, 13].

To leverage all the available data, we investigate more flexible approaches that do not limit the selection of peptides based on their source, length, and presenting MHC allele. Based on our data analysis, we show how the heterogeneity of the resulting data set can be viewed as a multi-domain structure, which motivates the application of different transfer learning techniques in combination with the flexible transformer architecture [14]. In contrast to the domain adaptation setting with one source and one target domain [15], we consider several domains and aim to improve the performance on each domain by leveraging training data from all domains.

We investigate how the prediction performance on individual domains is affected by including data from the other domains in the training process, where improved performance is denoted as positive transfer and reduced performance as negative transfer [16].

For all empirical studies, it is critical to keep in mind that having several underlying domains with varying fractions of positives entails the risk of shortcut learning [17–19], that is, using domain-specific features to classify peptides based on their domain identity instead of T-cell response-specific patterns. To obtain shortcut-invariant model performance estimates, we thus propose a domain-aware evaluation scheme.

Our results indicate that shortcuts based on peptide sources and MHC alleles are indeed learned by the transformer and lead to inflated performance estimates when the domain-aware evaluation is not applied. We further observe that per-source fine-tuning is effective in enabling positive transfer across a wide range of peptide sources and show that our final model is competitive with existing state-of-the-art approaches for predicting T-cell responses for human peptides.

The rest of the manuscript is organized as follows. In the next section, we first explain the data set construction and analyze the domain structure in the data, which motivates the application of transfer learning. Based on these observations, we describe the baseline transformer model of our studies, followed by the two main ideas we build on top of it: adversarial domain adaptation and per-source fine-tuning. We also extensively describe the domain aware evaluation scheme. In the empirical part, we evaluate the practical impact of shortcut learning, followed by an evaluation of the two transfer learning techniques. In the last section, we discuss the lessons learned and limitations of our study.

## Materials and methods

We first describe the T-cell response data set construction and analyze the domain structure in the data. Then follows an introduction of the baseline transformer model for T-cell response prediction. To account for the identified domain structure, we describe two transfer learning learning approaches and the domain-aware evaluation scheme.

### Construction of a T-cell response data set

We use the Immune Epitope Database^1^ (IEDB) [9] to construct a T-cell response data set. A data point consists of the amino acid sequence of the peptide, a binary label representing the T-cell response in the form of IFN$\gamma$, the allele of the MHC molecule that presented the peptide to a T cell, the class of the MHC allele (I or II), and the source of the peptide. The source can be an organism or a virus.

For some peptides in the data set, the MHC alleles are not specified in the IEDB. Additionally, the MHC allele information is incomplete since one peptide can often be presented on several MHC alleles. However, not all combinations of peptides and MHC alleles are tested. Since the existing MHC information in the IEDB is mostly based on predictions, we use existing models to obtain a list of MHC alleles for each peptide in a consistent way. NetMHCpan 4.1 [4] serves as a predictor for peptides with MHC class I alleles, and NetMHCIIpan 4.0 [4] for peptides with MHC class II alleles. We select the 100 most frequent alleles, which account for 98.7% of peptide-MHC combinations in the IEDB data set, and use all MHC class-matched combinations of peptides and MHC alleles as input for the two predictors.

We assign an allele to a peptide if it is predicted to be a weak binder. NetMHCpan 4.1 and NetMHCIIpan 4.0 define a peptide as a weak binder if its prediction score is within the top 2% of prediction scores for random natural peptides. Peptides that are predicted to not bind to any of the alleles get assigned a default allele for the respective MHC class. This applies to 29.7% of class I peptides and 66.5% of class II peptides. Since the MHC alleles are only relevant for us to identify MHC allele-specific patterns in peptides, the relatively large fraction of default alleles is not problematic because peptides with a default allele likely have no strong allele-specific patterns. Table 1 shows the resulting statistics about the MHC classes, T-cell response labels, and peptide lengths of the T-cell response data.

**Table 1:** Statistics of the data set. The columns for positives/negatives refer to the number of peptides with the respective T-cell response label

	MHC alleles	Positives	Negatives	Pep. lengths
MHC I	54	4460	12,697	8–15
MHC II	46	10,247	8207	9–25
Total	100	14,707	20,904	8–25

### Domain structure in T-cell response data

**Fig. 1:**
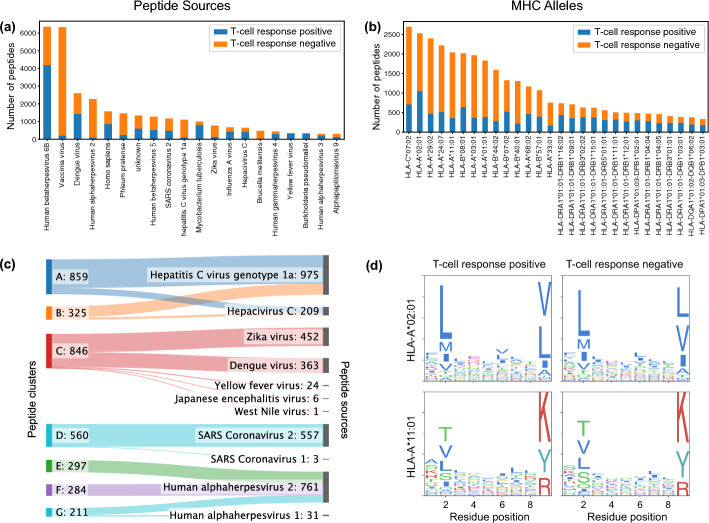
Domain structure of T-cell response data. **a** Distribution of T-cell response positives and negatives per peptide source. **b** Same plot for MHC alleles. **c** Clusters (indexed with A-G) of similar peptides (left) and the sources of peptides the clusters consist of (right). Numbers show peptide counts. **d** Sequence logos for the MHC alleles HLA-A*02:01 (first row) and HLA-A*11:01 (second row). The columns represent T-cell response positives (left) and negatives (right)

Based on the data analysis presented in Fig. 1, we identify two domain structures in the T-cell response data set: peptide sources (left column) and MHC alleles (right column). The following paragraphs describe these two domain structures in more detail.

Figure 1a shows that the peptides in the T-cell response data set originate from several sources and that the fraction of T-cell response positives varies strongly between sources. When studying T-cell responses for one peptide source, a protein of that source is often screened by testing a set of largely overlapping peptides that originate from the protein. These overlaps lead to clusters of peptides that make peptides within one source more similar than peptides from a different source.

Motivated by the size of the binding core in peptides [20], we define that peptides correspond to the same cluster if they share a sub-sequence of length nine. The Sankey diagram in Fig. 1c confirms that the seven largest peptide clusters typically consist of peptides from only a few sources.

In analogy to the distribution of peptide sources in Fig. 1a, Fig. 1b shows the distribution of MHC alleles. As peptides can be associated with several MHC alleles, we show a subset of MHC alleles that together cover a large fraction of peptides. Compared to peptide sources, there is a lower variability in the fraction of T-cell response positives between MHC alleles.

The distinct binding motifs of MHC alleles [10] introduce an MHC-based domain structure in T-cell response data. We visualize the sequence logos for two exemplary MHC alleles and peptides of length nine, separately for T-cell response positives and negatives, in Fig. 1d. For comparing two sequence logos, we further calculate the average of the position-specific Jensen-Shannon divergence (JSD) of the underlying weight matrices, a common metric to quantify the difference among sequence logos [21, 22].

At the anchor positions two and nine, there are obvious differences between the MHC alleles in the sequence logos and thus the average JSDs are high for comparing both positive logos (0.186) and both negatives (0.171). However, visually comparing sequence logos between T-cell response positives and negatives does not reveal obvious differences for either MHC allele, which is supported by substantially smaller average JSDs of 0.015 for HLA-A*02:01 and 0.030 for HLA-A*11:01.

Since studies of T-cell responses often focus on just one peptide source and one MHC class or only consider peptides that are presented on certain MHC alleles, the joint distribution of peptide sources and MHC alleles is not uniform (see Supplement S1). Consequently, peptide sources can also feature the patterns of the MHC alleles with which they co-occur.

**Fig. 2:**
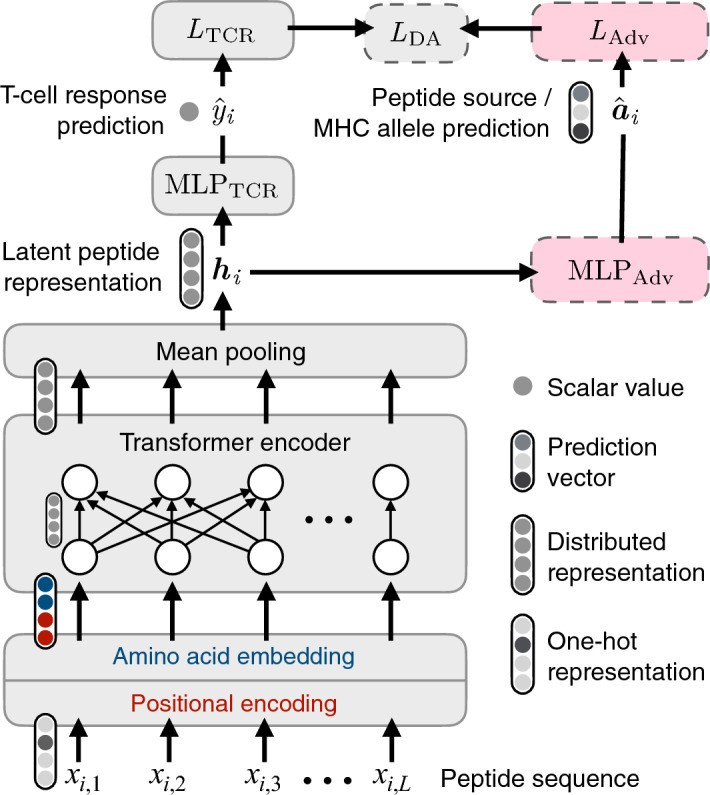
Model architecture for T-cell response prediction. Shading of boxes indicates with which objectives the components are trained. Boxes with dashed borders are only used in the adversarial domain adaptation setting

### Transformer model

We use the transformer architecture [14], as shown in Fig. 2, to capture T-cell response-specific patterns in peptide sequences. To apply the transformer, we formalize the data set to consist of data points $\{(\boldsymbol{x}_i, y_i)\}_{i=1}^{N_{\text {data}}}$, where $\boldsymbol{x}_i = (x_{i,1}, \dots , x_{i,L})$ is the *i*-th peptide sequence with each of the $x_{i,l}$ representing one of 20 amino acids or a padding token. The padding token allows the sequences to all have the same length *L*, which corresponds to the maximal peptide length. We denote the binary label indicating whether the peptide $\boldsymbol{x}_i$ leads to a T-cell response with $y_i$.

Our model gets a one-hot representation of the peptide sequence $\boldsymbol{x}_i$ as input. Then the model maps each amino acid or padding token $x_{i,j}$ of the peptide sequence to a learned embedding $\boldsymbol{e}_{i,l} \in \mathbb {R}^{d/2}$. To tell the transformer the positions at which the amino acids occur in the peptide, we define a learned embedding $\boldsymbol{p}_l \in \mathbb {R}^{d/2}$ for each position $l \in \{1, \dots , L\}$. Additionally, we use positional encodings $\boldsymbol{s}_l \in \mathbb {R}^{d/2}$ based on sine functions with varying frequencies (see [14] for more details). We then add the two variants of positional vectors and concatenate them with the amino acid embeddings to obtain a vector representation $$\left( {\begin{smallmatrix} \boldsymbol{e}_{i,l} \\ \boldsymbol{p}_l + \boldsymbol{s}_l \end{smallmatrix}}\right) \in \mathbb {R}^{d}$$ for each element $x_{i,l}$ of the peptide sequence $\boldsymbol{x}_i$. We note that the described encoding is only one among many options, see Supplement S4 for the description of alternatives and a comparative evaluation.

The resulting sequence of vector representations is the input of a transformer encoder layer, which updates the vector representations based on interactions between amino acids using a mechanism called multi-head self-attention (see [14] for more details).

As the output of the transformer encoder is again a sequence of vector representations, we apply mean pooling to obtain a single vector $\boldsymbol{h}_i \in \mathbb {R}^{d}$, which is a latent representation of the *i*-th peptide. In a similar way as described here, the transformer architecture has been used in earlier work to learn representations of peptides [6] and proteins [23].

Using the latent peptide representation $\boldsymbol{h}_i$ as input, a multilayer perceptron (MLP) with one hidden layer of size *d* computes the T-cell response prediction $\hat{y}_i = \textsc {MLP}_{\text {TCR}}(\boldsymbol{h}_i)$.

All described components are trained to minimize the loss

1 $$\begin{aligned} L_{\text {TCR}}= \frac{1}{N_{\text {data}}} \sum _{i=1}^{N_{\text {data}}} \text {CE}(y_i, \hat{y}_i), \end{aligned}$$

with the binary cross-entropy between T-cell response labels and predictions being defined as

2 $$\begin{aligned} \text {CE}(y_i, \hat{y}_i) = -(y_i \text {log}(\hat{y}_i) + (1-y_i)\text {log}(1-\hat{y}_i)). \end{aligned}$$

In the empirical studies, we refer to the transformer model baseline described in this section as BASE-T.

### Adversarial domain adaptation

As described above, the T-cell response data set has an underlying domain structure of peptide sources and MHC alleles. This heterogeneity suggests that predictions might be biased by the class imbalance (see Fig. 1a, b). Additionally, domain-specific features might be used by a machine learning model instead of more robust features that are independent of MHC alleles and peptide sources.

To address these issues, we apply adversarial domain adaptation [24] to encourage the model to perform predictions independently of the domain of a peptide. Our multi-domain setting is different from the classic domain adaptation setting with one source and one target domain. We thus use a variant of adversarial domain adaptation that is very similar to *multi-domain adversarial learning* [25].

The model components for implementing adversarial domain adaptation are marked with dashed borders in Fig. 2. To encourage domain invariance, we define a new training objective:

3 $$\begin{aligned} L_{\text {DA}}= L_{\text {TCR}}- \lambda L_{\text {Adv}}. \end{aligned}$$

As before, minimizing $L_{\text {TCR}}$ is necessary for performing the main task, T-cell response prediction. The term $-L_{\text {Adv}}$ measures how much information about the domain identities is encoded in the latent peptide representations $\boldsymbol{h}_i$. The strength of domain adaptation is determined by the hyperparameter $\lambda$. In the following, we explain adversarial domain adaptation considering peptide sources as domains. To capture how much information about peptide sources is encoded in $\boldsymbol{h}_i$, we train an adversarial network $\textsc {MLP}_{\text {Adv}}$ with input $\boldsymbol{h}_i$ and output $(\hat{a}_{i,1}, \dots , \hat{a}_{i,N_{\text {source}}})^{\intercal } = \hat{\boldsymbol{a}}_i \in \mathbb {R}^{N_{\text {source}}}$ to predict how likely peptide *i* originates from each of the $N_{\text {source}}$ peptide sources.

$\textsc {MLP}_{\text {Adv}}$ is trained concurrently with the other parts of the T-cell response model using the following multi-label classification objective $L_{\text {Adv}}$, where $a_{i,j}$ are the ground truth peptide source labels:

4 $$\begin{aligned} L_{\text {Adv}}= \sum _{i=1}^{N_{\text {data}}} \; \sum _{j=1}^{N_{\text {source}}} \text {CE}(a_{i,j}, \hat{a}_{i,j}). \end{aligned}$$

While the parameters of $\textsc {MLP}_{\text {Adv}}$ are trained to minimize the peptide source classification error, $L_{\text {Adv}}$ is re-used with a negative sign in $L_{\text {DA}}$. This means that latent peptide representations produced by the transformer are encouraged to confuse $\textsc {MLP}_{\text {Adv}}$ by making latent representations from different domains more similar, suggesting the name *adversarial* domain adaptation. The above explanations for peptide sources can be directly generalized to MHC alleles. In the empirical studies, we refer to the approach described in this section as ADA-T.

### Per-source fine-tuning

When one machine learning model is trained on data from several domains, the performance on individual domains can be reduced compared to models that are trained on data from one domain only. Such instances of negative transfer are more likely to occur if domains are too dissimilar [16]. Negative transfer is also possible when applying adversarial domain adaptation because there is only one model for all domains and data points from all domains are encouraged to be represented similarly in the model. To avoid negative transfer, a transfer learning technique is needed that provides more flexibility to adapt to individual domains, while still allowing positive transfer from other domains.

As an alternative to adversarial domain adaptation, we propose to pre-train a T-cell response model until convergence on all domains and then continue training on only one domain. This second step is also called fine-tuning. We allow all model parameters to be updated during fine-tuning such that the model has the necessary flexibility to correct instances of negative transfer. In the empirical studies, we refer to this approach as FINE-T. This transfer learning technique is commonly applied in combination with the transformer and proteins [23] or peptides [6].

### Bag of amino acids baseline

To allow a direct comparison of our proposed transformer-based models to a simpler model that is trained on the same data set, we use a *bag of amino acids* (Bag-Of-AA) baseline, since it can be viewed as a simplified version of PRIME [7].

This baseline model counts how often each of the 20 amino acids occurs in a given peptide and puts the frequencies into a 20-dimensional vector. An MLP with one hidden layer receives these vectors as input and is trained to predict the T-cell responses. As with the transformer models, we use the binary cross-entropy as training objective. The Bag-Of-AA model does not consider positional information, that is, it does not see at which positions the amino acids occur in the peptide. The amino acid frequencies allow the model to capture possible preferences for certain amino acids that T cells might have.

### Performance evaluation

We measure the model performance with the area under the ROC curve (AUC) based on the ground-truth T-cell response labels $y_i$ and the predictions $\hat{y}_i$.

We partition the data set $\mathcal {D}$ into five disjoint subsets $\mathcal {D}_{1},\dots ,\mathcal {D}_{5}$, and then permute and re-combine them into training, validation, and test sets. This setting ensures that the partitions are large enough to each have a representative distribution of the domains.

Within a peptide cluster, sequences can be nearly identical (cf. Section Domain structure in T-cell response data). Splitting the data completely at random could lead to such near identical sequences being distributed among training, validation, and test sets. This can potentially cause inflated generalization estimates. To prevent this, we assign peptide clusters as a whole (instead of single peptides) to a randomly selected partition [20].

Throughout this manuscript, we carry out all method development and hyperparameter tuning on the union of the first four data subsets. Using $\mathcal {D}_1,\dots ,\mathcal {D}_{4}$ as input for a four-fold cross validation, we refer to the resulting mean AUC over the four folds as *validation performance*. This leaves $\mathcal {D}_{5}$ as unseen, independent test data set for evaluating generalization capabilities in the final study, where we refer to the evaluation of the predictions on $\mathcal {D}_{5}$ while training on $\mathcal {D}\setminus \mathcal {D}_{5}$ as *test performance*.

Since this test performance is only a single number, we also carry out a nested cross validation that uses the previous experiment as its first outer loop and additionally computes test evaluations for $D_j$ for $j\in \{1,\dots ,4\}$. Training on $\mathcal {D}\setminus \mathcal {D}_{j}$ in the outer loop always involves the re-tuning of numeric hyperparameters via an inner four-fold cross-validation. We note, however, that more general choices regarding the model architecture and the range of tunable hyperparameters originate from the primary validation study carried out on $\mathcal {D}\setminus \mathcal {D}_5$. Hence, this approach permits to calculate an uncertainty quantification of the test performance in terms of standard errors at the cost of a small potential for test data leakage.

### Shortcut detection and domain-aware evaluation scheme

The domain structure with different T-cell response positive/negative ratios per domain (see Section Domain structure in T-cell response data) suggests that *shortcuts* can be learned to predict T-cell responses by first picking up domain-specific features to identify domains and then outputting the majority class within the domain.

We ensure that our evaluation is not influenced by the use of MHC allele-based or peptide source-based shortcuts by evaluating our models on subsets of the data containing only one MHC allele and one peptide source. Shortcuts in the form of predicting the majority class of a peptide source or an MHC allele do not provide predictive power within such subsets. The performance on these subsets thus represents what a model has learned beyond the shortcuts.

When summarizing the per-subset results to an overall score, we ensure that every peptide has the same influence on the overall score, even if it occurs in several subsets as a consequence of being presented on several MHC alleles.

To describe our domain-aware evaluation setup in more detail, we introduce the following formalization: Let $(G_k)_{k=1,\dots ,n} \, (n \le \#\text {alleles} \cdot \#\text {sources})$ be the list of peptide groups. Each peptide group $G_k$ corresponds to an allele-source combination. A group $G_k$ contains indexes of peptides from only one source and one allele. For the i-th peptide in our data set, we denote the T-cell response label as $y_i$, the prediction as $\hat{y}_i$ and corresponding lists for a peptide group *G* as $\boldsymbol{y}_G = (y_i)_{i \in G}$ and $\boldsymbol{\hat{y}}_G = (\hat{y}_i)_{i \in G}$.

To achieve that every peptide has the same influence on the overall AUC score, despite being present in varying numbers of groups, we define peptide weights $w_i$ to be the inverse of the number of groups the peptide is part of: $w_i = \frac{1}{|\{G_k \mid i \in G_k \}|}$. We define the total weight of peptides associated with a group as $w_G = \sum _{i \in G} w_i$. Analogously to the labels and predictions, we denote the list of peptide weights for a group *G* as $\boldsymbol{w}_G = (w_i)_{i \in G}$.

The AUC score that is adjusted for both source and allele-based shortcuts can now be defined as follows:

5 $$\begin{aligned} \text {AUC} = \sum _{k=1}^n \frac{w_{G_k}}{\sum _{k=1}^n w_{G_k}} \cdot \texttt {roc\_auc\_score}(\boldsymbol{y}_{G_k}, \boldsymbol{\hat{y}}_{G_k}, \text {sample\_weight}= \boldsymbol{w}_{G_k}) \end{aligned}$$

We use the roc_auc_score function from Scikit-learn [26]. Weighting the per-group AUC scores with the normalized per-group weights accounts for both varying group sizes and for peptides being part of varying numbers of groups.

To obtain the *allele adjusted* results, we define the peptide groups based on MHC alleles only, s.t. each group contains peptides from only one allele. Then we apply the same weighting scheme as above. This setting only adjusts for shortcuts based on MHC alleles.

To obtain the *source adjusted* results, we define the peptide groups based on their sources, s.t. each group contains peptides from only one source, and apply the weighting scheme as above. This setting only adjusts for shortcuts based on the organism or virus the peptide originates from.

## Results

The empirical studies in this article seek answers to five research questions.

1. Does the domain structure in the data lead to shortcut learning?
2. Can shortcut learning be reduced while improving adjusted performance?
3. Are there instances of negative transfer between the domains?
4. Is there also positive transfer and can it be leveraged?
5. How does our final approach compare to other tools for human peptides?

Each question corresponds to one of the following subsections and the answer to each question serves as motivation for the next one in the list. A detailed summary of all studies carried out and how they relate to each other is given in Supplement S2. We provide details on the implementation and the model hyperparameters in the Supplement S3.

**Fig. 3:**
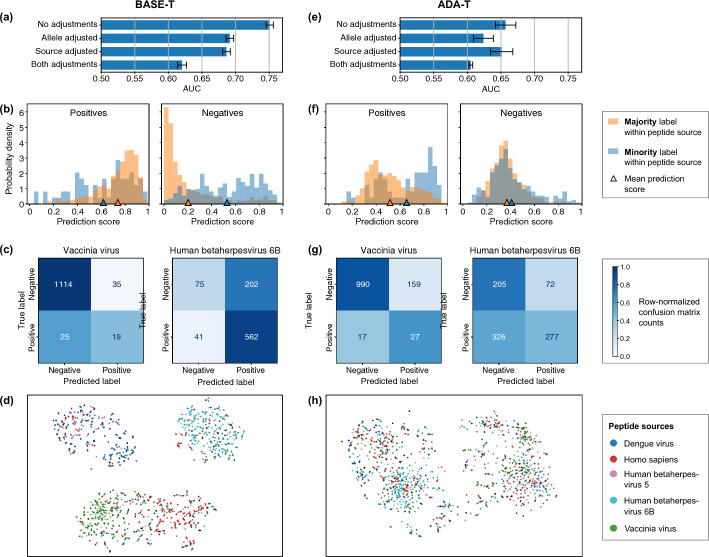
Shortcut learning and the effect of adversarial domain adaptation. The left column shows results for BASE-T and the right column shows corresponding results for ADA-T with adversarial domain adaptation being applied on peptide sources. **a+e** Model performance on validation data with different settings of accounting for shortcuts in the evaluation. For the "allele adjusted" performance, peptides are grouped by MHC alleles in the evaluation to detect shortcuts based on MHC alleles. "Source adjusted" is analogous for grouping peptides by their source. **b+f** Distribution of prediction scores, separated by the T-cell response labels. For each label, two distributions are shown that correspond to the two most frequent peptide sources, Human betaherpesvirus 6B (majority of labels is positive) and Vaccinia virus (majority of labels is negative). The markers on the x-axis show the mean prediction scores of the two distributions. **c+g** Confusion matrices derived from the prediction scores for the two peptide sources in **b+f** by applying a prediction threshold of 0.5. **d+h** t-SNE visualization of the latent peptide representations $\textbf{h}$

### Shortcut learning

We investigate the prevalence of shortcut learning in the baseline T-cell response prediction model, BASE-T, trained on the full T-cell response data set, that is, on all peptide sources and MHC alleles (MHC I+II) combined. The results are summarized in the left column of Fig. 3.

The prediction performance in terms of AUC  with and without shortcut adjustment in the evaluation is shown in Fig. 3a. We observe that the shortcut adjustment w.r.t. alleles and peptide sources leads to an absolute AUC  decrease of 0.06 when applied separately, and a further decrease by 0.07 when combined, which indicates that the model learns shortcuts based on both MHC allele-specific and peptide source-specific features. We also test whether these effects depend on the specific positional encoding (see Methods), but find this not to be the case (Supplement S4).

To verify that shortcuts are actually based on the label imbalance within different domains (see Figs. 1a and 1b), we plot the prediction scores of peptides from the two most frequent peptide sources, Human betaherpesvirus 6B (mostly positives) and Vaccinia virus (mostly negatives) as histograms, separated according to the true response labels in Fig. 3b. Additionally, we show the corresponding confusion matrices in Fig. 3c.

We find that the distribution of prediction scores is always shifted towards the majority class within the peptide source: Human betaherpesvirus 6B peptides with a positive label tend to get higher prediction scores than Vaccinia virus peptides with a positive label. For negatives, the Vaccinia virus peptides get lower prediction scores than Human betaherpesvirus 6B peptides. The shift towards the majority label is also apparent in the confusion matrices, where most of the labels in the minority class are wrongly predicted.

In addition to the analysis of model outputs, we investigate the internal representations of the models regarding indications of shortcut learning. To use shortcuts based on peptide sources, we expect BASE-T to learn source-related features in the latent peptide representations *p*.

To test whether this is the case, we use t-stochastic neighborhood embedding (t-SNE) by [27] to project the high-dimensional latent peptide representations *p* to two-dimensional points. We use the t-SNE implementation of the TensorFlow embedding projector with the default parameters for learning rate and perplexity.

The cluster structure in the t-SNE plot of latent peptide representations in Fig. 3d provides indeed further evidence that features related to peptide sources are learned by BASE-T. For example, the Human betaherpesvirus 6B peptide representations (cyan) concentrate mostly in the top right corner while the Vaccinia virus peptides (green) can be found mostly in the lower left corner.

We thus answer the first research question posed above in the affirmative: a baseline T-cell response prediction model can indeed exhibit a substantial amount of shortcut learning due to the domain structure in the data.

### Adversarial domain adaptation

In order to study whether this shortcut learning can be prevented by encouraging domain invariance in the model architecture and whether this constraint could serve as regularizer to improve the model performance, we repeat the previous study with ADA-T, the adversarial domain adaptation approach. We set the domain adaptation strength of ADA-T to $\lambda =10$ based on the hyperparameter experiment described in Supplement S5. The results are summarized in the right column of Fig. 3.

In Fig. 3e we observe that in contrast to BASE-T, the performance of ADA-T without adjustments and with source shortcut adjustment is nearly identical, which demonstrates that adversarial domain adaptation is effective in reducing shortcut learning based on peptide sources. When adjusting for both possible shortcuts in the evaluation, ADA-T leads to slightly reduced predictive performance compared to BASE-T.

In Fig. 3f we observe that ADA-T overcorrects the bias towards the majority label within sources for the T-cell response positives, as the Vaccinia virus peptides (mostly negatives) now get even higher prediction scores than Human betaherpesvirus 6B peptides. For the T-cell response negatives, the distributions of prediction scores corresponding to the two peptide sources are now well aligned, indicating that no source shortcuts are used. Considering the confusion matrices in Fig. 3g, most of the labels in the minority classes are correctly predicted by ADA-T. These findings may also explain the slight drop in overall prediction accuracy of ADA-T observed in Fig. 3e, as the number of correct predictions in the minority classes is increased at the expense of additional errors in the majority classes.

We also carried out this study for other peptide sources (Supplement S6), where a similar effect is visible for most of them, albeit to a smaller degree, which is likely due to the smaller sample size. Finally, we observe in Fig. 3h that the t-SNE plot is less clustered for ADA-T, demonstrating that source-related features are removed from the peptide representations.

We thus answer the second research question in the negative: while ADA-T does indeed eliminate shortcut learning based on the peptide sources, this does not translate to an improvement in predictive performance.

### Negative transfer between domains

Next, we study this phenomenon in more detail by investigating why applying adversarial domain adaptation to peptide sources does not improve performance despite encouraging transfer between the sources.

Considering a peptide from one source, very similar peptides in terms of Levenshtein distance can be found with a higher likelihood within that source than from the other sources (Supplement S7). This motivates our hypothesis that there is negative transfer between sources as peptide sequences from different sources are too dissimilar.

To test this hypothesis, we compare BASE-T, being trained on all peptide sources and MHC alleles (*multi-domain* setting), with a combination of BASE-T models that eliminate transfer between sources and alleles respectively. All evaluations apply the full shortcut adjustment.

In a *per-source* setting, we train separate BASE-T models for every peptide source, each of which contains peptides from several MHC alleles. To make the performance of the models in the *per-source* setting directly comparable to the *multi-domain* setting, we first aggregate the predictions for each source (performed by different models) into one data set again. Then we apply the evaluation on groups of peptide source/MHC allele combinations exactly as in the *multi-domain* setting. This evaluation also ensures that only predictions coming from the same per-source model are compared to each other because AUC values are computed per peptide source/MHC allele combination. Conversely, the *per-allele* setting is a combination of separate BASE-T models for each MHC allele, each of which is trained on data from several peptide sources.

The corresponding validation study (Supplement S8) shows that the AUC  of the multi-domain model is reduced by 0.05 compared to the per-source models and by 0.02 compared to the per-allele models, confirming our hypothesis that there is negative transfer between peptide sources and, to a smaller degree, between MHC alleles.

**Fig. 4:**
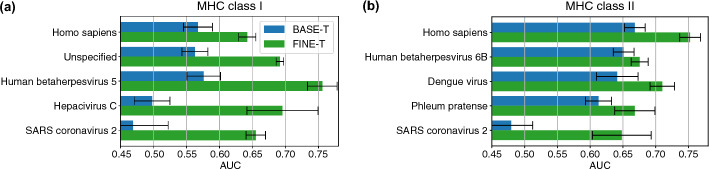
Validation performance of BASE-T and FINE-T models for several peptide sources. Results for MHC I are shown in **a** and for MHC II in **b**. For each MHC class, the five most frequent peptide sources are selected. Peptide sources with only positives or only negatives in one of the test data partitions are excluded

### Per-source fine-tuning

While negative transfer mainly occurs between sources and per-source models achieve the highest accuracy in predicting T-cell responses, we investigate whether there is also potential of positive transfer between peptide sources. By training BASE-T models and excluding one peptide source while evaluating on the excluded peptide source, we analyze possible transfer to unseen sources. This experiment (Supplement S9) shows that BASE-T performs better than chance for most of the unseen peptide sources, despite a drop in performance compared to the setting with seen sources. The drop in performance is in line with the negative transfer we have described above, while the better-than-chance generalization suggests potential of positive transfer between sources.

To leverage this potential, we explore another transfer learning technique. We initially train BASE-T on all sources and subsequently fine-tune per-source models with model parameters initialized using BASE-T. With this per-source fine-tuning approach, which we refer to as FINE-T, we aim to combine the advantages of per-source models with the possibility of positive transfer between sources. The results for the five most frequent peptide sources are shown, separated according to MHC class, in Fig. 4.

FINE-T achieves higher AUC  values than BASE-T for all ten source/MHC combinations. We also observe that the difference is stronger for MHC I peptides than for MHC II peptides, which may be due to more room for improvement, as BASE-T performs worse for MHC I compared to MHC II peptides.

We additionally carried out the same study for less frequent peptide sources, i.e., all that permit at least one positive and one negative instance per cross validation test fold (SupplementS10). We observe that FINE-T performs always at least as well base BASE-T, and in most cases, it is strictly better. For very rare peptide sources, the performance of both models is not better than random, though.

We also studied the performance of separate per-source models without fine-tuning. The resulting AUC values (see Supplement S11) are overall between BASE-T and FINE-T, suggesting that the combination of both ideas, per source models and fine-tuning, is responsible for the superior performance of FINE-T. By generating many random permutations of the T-cell response labels, we also verify that FINE-T learns a signal that cannot be explained by random structure in the data (see Supplement S12).

We answer the fourth research question posed above in the affirmative: positive transfer between sources is possible, and it can be leveraged by the per-source fine-tuning method. FINE-T is thus the final model of our study and warrants a comparison with existing tools.

### Comparison with existing tools

T-cell response predictions for peptides from human proteins are of special interest for the development of personalized cancer vaccines since neo-antigens also originate from human proteins [28]. Thus, we focus our final analysis on human peptides and compare the performance of FINE-T, which was the best model in the validation study, to existing pre-trained models from the literature.

**Fig. 5:**
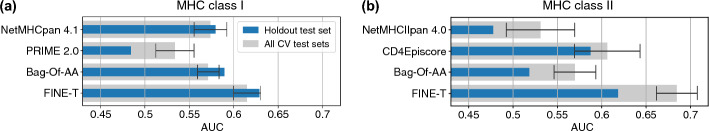
Test performance on human peptides presented on **a** MHC class I and **b** MHC class II. For both MHC classes, the first two rows correspond to existing models from the literature, which are trained on other data sets. The Bag-Of-AA baseline and FINE-T are trained on the same data. Blue bars correspond to the evaluation on the previously completely unused test set. Grey bars show the mean AUC from the final nested cross validation

NetMHCpan 4.1 and NetMHCIIpan 4.0 [4] have been trained to predict the presentation of peptides on MHC molecules. Despite not being trained on T-cell response data, we include these models since they are commonly used in the context of personalized cancer vaccines. PRIME 2.0 [7] has been trained on T-cell response data with MHC I neo-antigens, whereas CD4Episcore [8] has been trained on MHC II T-cell response data from several peptide sources. Since the training data for all pre-trained models deviates from our training data, we also include the Bag-Of-AA baseline, trained on the same data set as FINE-T, in the comparison. In contrast to our previous studies, we now train a model on the union of the first four data subsets, and evaluate on the previously unused data subset $\mathcal {D}_5$. In addition, we also carry out a full nested cross-validation. The results are shown in Fig. 5.

For MHC I peptides, FINE-T achieves with a value of 0.615 the highest AUC among all models. PRIME 2.0 yields the lowest performance, which is likely due to the differences in the training data. For MHC II peptides, FINE-T also performs better than existing approaches, and the AUC is, with a value 0.68 even slightly better than in the validation study. While is has been observed in previous work that combining MHC binding predictions from several alleles has a predictive value for immunogenicity [29], on the data set we use, this applies only to MHC I (NetMHCpan 4.1) but not to MHC II (NetMHCIIpan 4.0). The large performance difference between the Bag-Of-AA model and FINE-T suggests that the possibility to use positional information of amino acids in the peptide sequence is indeed beneficial, and more so for MHC class II compared to MHC class I.

## Discussion

In this work, we studied the effect of various transfer learning techniques on the problem of T-cell response prediction. We have observed that predicting T-cell responses to peptides is a challenging problem not only due to the limited sample size, but also since the data features a multi-domain structure, which entails, as illustrated in this article, the danger of shortcut learning.

To ensure that performance estimates are not inflated by the use of shortcuts, we have defined a domain-aware evaluation scheme. We have then demonstrated that shortcut learning can be effectively reduced by applying adversarial domain adaptation in an approach dubbed ADA-T. This shortcut reduction did, however, not directly improve predictive performance, which is likely due to large domain differences and negative transfer between domains.

To enable positive transfer while preventing negative transfer, we have pre-trained a transformer model on all data domains and then created fine-tuned models for individual domains, resulting in a model dubbed FINE-T. This approach improved predictive performance considerably and performs slightly better than several existing methods, although the overall performance of all models still leaves room for improvement.

We thus conclude that the overall effect of transfer learning as a broad strategy on T-cell response prediction is mixed, with FINE-T showing benefits, whereas ADA-T being not helpful. Beyond raw predictive performance, there are a few other points to consider, though.

Since modeling the binding of individual T-cell receptors (TCRs) and peptide:MHC complexes is difficult and does not directly capture the T-cell response [30–32], many prediction models, including ours, assume general patterns in peptide sequences that make peptide recognition more likely in many individuals [11, 33]. This assumption is based on the public TCR repertoire concept of antigen-recognizing TCRs being likely shared among individuals [34].

Once more data of paired TCR and peptide:MHC complexes become available and protein structure predictions are further improved, the public TCR repertoire assumption might be relaxed. Modeling the interaction between individual TCRs and peptide-MHC complexes has the potential to improve T-cell response prediction in principle [35].

Many machine learning models that can reasonably predict which TCRs bind to a given, fixed peptide:MHC complex have been suggested and benchmarked in the literature [35, 36]. Recently, several models have been suggested that aim to predict TCR binding to peptide-MHC complexes for peptides unseen during training [37–39]. This more challenging setting with variable peptide sequences is closer to the T-cell response problem we have studied. In an independent study on a new data set with cancer neo-antigens, these models perform close to chance, while they perform better than chance on a test set dominated by viral epitopes [40]. This result suggests that peptide source biases are not only a challenge in the T-cell response prediction task we considered but also in the related TCR binding prediction problem.

One potential limitation of our study arises from the fact that a fraction of the peptides are assigned the default MHC allele due to their lack of strong MHC allele-specific patterns. Potential shortcut learning within the group of peptides with the default allele is not corrected for with our domain-aware evaluation scheme. However, peptides with the default allele are expected to be less prone to shortcut learning. To obtain MHC allele predictions, we use the NetMHCpan/NetMHCIIpan models, which are explicitly trained on MHC allele prediction. Given that NetMHCpan and NetMHCIIpan do not pick up strong MHC allele-specific patterns in some peptides, it is unlikely that T-cell response models can capture such patterns and use them as shortcuts.

In a broader context, the present study demonstrates the importance of knowing possibly hidden underlying structures in the training data for two reasons. First, the risk of shortcut learning is well known in domains like image recognition or natural language processing [19]. However, in biological sequence data, invalid features might be harder to discover since they can be encoded in more complex patterns spanning only specific positions in sequences, as it is the case for anchor positions in peptides (see Fig. 1b). Second, knowing about the heterogeneity of a data set and viewing it as a multi-domain structure can motivate the use of transfer learning techniques. This transfer learning setting is also referred to as multi-domain learning in previous work [25, 41]. As we have observed with ADA-T and as it is also reported for other tasks such as classification of images, texts, or molecules [42], transfer learning methods like adversarial domain adaptation can sometimes decrease the classification performance. Similarly, caution has to be applied in the data selection process since increasing a data set at the cost of more heterogeneity can lead to instances of negative transfer [12].

In a similar direction, it is noteworthy that to this date, all approaches for T-cell response prediction use their own specific data selection criteria, differing, for example, in the selected assays, measured cytokines, or the considered peptide sources [7, 8, 43]. We think that a benchmark data set that is well characterized in terms of its underlying structure, together with an evaluation scheme that is invariant to possible instances of shortcut learning, would be beneficial to facilitate objective development and a fair comparison of T-cell response prediction methods.

## Additional file

Supplementary file 1 (pdf 1205 KB)

## Data Availability

Data, source code, and trained models are available under https://github.com/JosuaStadelmaier/T-cell-response-prediction.

## Footnotes

1 https://www.iedb.org.

## Code availability

Source code and trained models are available under github.com/JosuaStadelmaier/T-cell-response-prediction.
