# Supplementary material for: Transfer learning for T-cell response prediction
Source: BMC Bioinformatics. 2026 Jun 10;27:124. doi: 10.1186/s12859-026-06492-2 (PMC13251289; doi:10.1186/s12859-026-06492-2)
Supplement: Supplementary file 1 — (pdf 1205 KB) [file 12859_2026_6492_MOESM1_ESM.pdf]

Transfer Learning for T-Cell Response Prediction

Supplementary Material

Josua Stadelmaier, Brandon Malone and Ralf Eggeling

S 1 Joint distribution of MHC alleles and peptide sources

The following heatmap shows the most frequent peptide sources and a representative subset of MHC alleles. As mentioned in Section 2.2, the patterns in the heatmap reveal that the joint distribution of MHC alleles and peptide sources is not uniform.

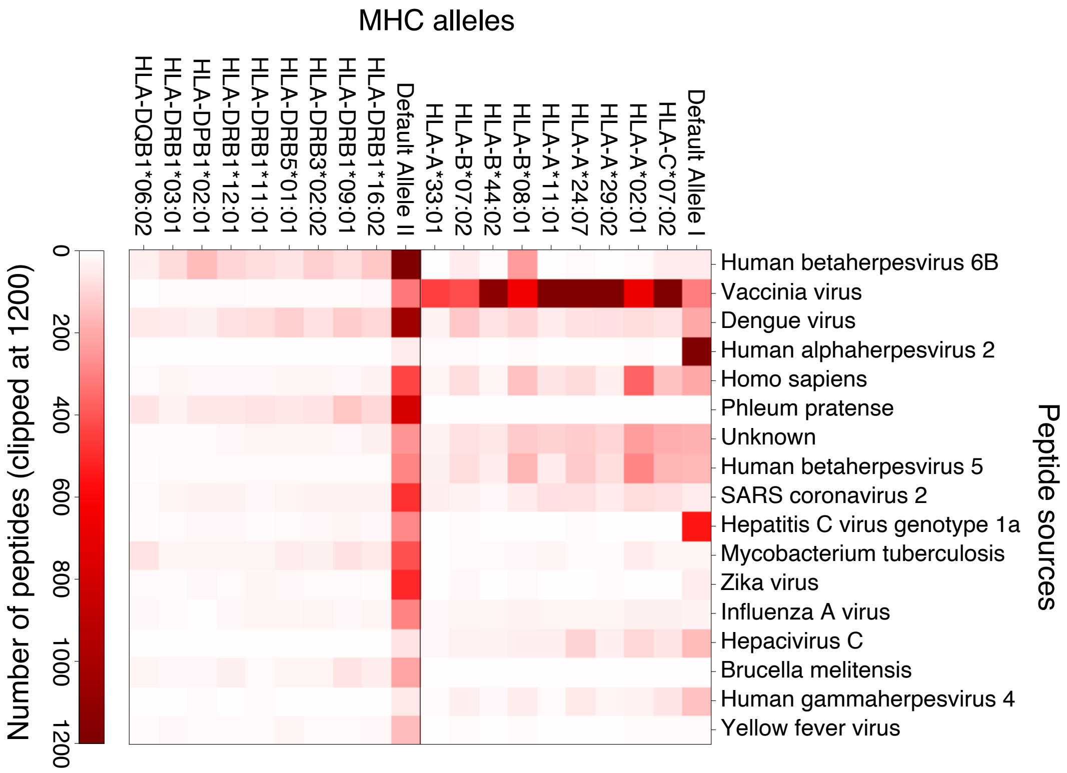

S 2 Overview of all main experiments

1) Shortcut learning: Does the domain structure in the data lead to shortcut learning?

Model	Train data	Evaluation setting	Cross-validation
BASE-T	All sources and alleles	No adjustments<br>Allele adjusted<br>Source adjusted<br>Both adjustments	Validation data

Result: Shortcuts are used by BASE-T.

2) Adversarial domain adaptation: Can shortcut learning be reduced while improving adjusted performance?

Model	Train data	Evaluation setting	Cross-validation
BASE-T<br>ADA-T	All sources and alleles	No adjustments<br>Allele adjusted<br>Source adjusted<br>Both adjustments	Validation data

Result: ADA-T reduces shortcut learning but does not improve performance, potentially due to neg. transfer.

3) Negative transfer between domains: Are there instances of negative transfer between the domains?

Model	Train data	Evaluation setting	Cross-validation
BASE-T	Subsets of<br>sources and alleles	Both adjustments	Validation data

Result: There is negative transfer between domains, especially between peptide sources.

4) Per-source fine-tuning: Is there also positive transfer and can it be leveraged?

Model	Train data	Evaluation setting	Cross-validation
BASE-T<br>FINE-T	All sources and alleles	Both adjustments	Validation data

Result: FINE-T improves performance over BASE-T.

5) Comparison with existing tools: How does FINE-T compare to other tools for human peptides?

Model	Train data	Evaluation setting	Cross-validation
FINE-T<br>Bag-Of-AA<br>several existing tools	All sources and alleles or<br>tool-specific data	Both adjustments	Test data

Result: FINE-T achieves the best performance among the evaluated tools.

Parameters that are compared in the corresponding experiment. (shaded cells: Evaluation setting in 1; Model and Evaluation setting in 2; Train data in 3; Model in 4; Model in 5)

S 3 Implementation and hyperparameter selection

For implementing the experiments, we use PyTorch and its built-in implementation of the transformer encoder. We perform gradient-based optimization using the stochastic optimizer Adam (Kingma and Ba, 2015). Besides regularization with dropout, we apply early stopping based on the validation performance with a maximum of 250 epochs.

To reduce the uncertainty of performance estimates resulting from the random initialization of network weights, sampling of mini-batches, and dropout, we repeat every experiment with different random seeds and then average the performance estimates.

We use a grid search combined with cross-validation to select suitable values for hyperparameters. Since exhaustively testing every hyperparameter combination of the transformer models in each experiment is computationally expensive, we perform an initial exploration of hyperparameters on all peptide sources and MHC alleles combined and then use smaller, more informed hyperparameter ranges for the individual experiments (see Table 1). Unless otherwise stated, we set the batch size to 500. The hyperparameter ranges for the Bag-Of-AA model are shown in Table 2.

The validation performance results from Section 3.1 to Section 3.3 are obtained by running the cross-validation on the first four data partitions $\mathcal{D}_1, \dots, \mathcal{D}_4$. The test results with standard error estimates from Section 3.4 are obtained by a nested cross-validation on all data partitions $\mathcal{D}_1, \dots, \mathcal{D}_5$.

Table 1: Hyperparameter ranges for the transformer models

Experiment	Learning rate	Dropout	Embedding dimension d	Attn. layers	Attn. heads
Initial exploration	{0.001, 0.01, 0.05}	{0, 0.1, 0.2, 0.3}	{16, 32, 64, 96}	{1, 2}	{4, 8, 16}
Sec. 3.1 / Fig. 3	0.005	0.2	64	1	8
ADA-T: Select $\lambda \in \{2, 4, \dots, 26\}$ as described in Section S 5.					
Sec. 3.2 / Supp. 2	0.01	{0, 0.1, 0.3}	32	1	8
Batch size set to 100 due to smaller data sets. Max epochs set to 100 for efficiency.					
Sec. 3.3 / Supp. 3	0.01	{0.1, 0.2, 0.3, 0.4}	{32, 48}	1	16
Sec. 3.4 FINE-T I	0.01	{0.1, 0.2}	{32, 48}	1	16
Sec. 3.4 FINE-T II	0.01	{0.2, 0.3}	{32, 48}	1	16

Table 2: Hyperparameter ranges for the Bag-Of-AA baseline

Experiment	Learning rate	Dropout	Hidden layer dimension
Sec. 3.4 Bag-Of-AA I / II	{0.01, 0.1, 0.2}	{0.1, 0.2}	{64, 96, 128}

S 3.1 NetMHCpan 4.1 and NetMHCIIpan 4.0 predictions

We include NetMHCpan 4.1 EL and NetMHCIIpan 4.0 EL to in our final test evaluation of T-cell response predictions for human peptides. With this, we aim to capture how much of the predictive power of the T-cell response models can be achieved with existing models of MHC presentation.

Paul *et al.* (2015) have observed that combining the MHC binding predictions for several alleles for a given peptide can provide immunogenicity estimates. Similar to the approach by Paul *et al.* (2015), we take the minimum of percentile rank scores over all MHC I or MHC II alleles in our data set to obtain estimates or T-cell responses.

S 4 Choice of positional encodings

We investigate the influence of different positional encodings of peptide sequences on the performance of the BASE-T model in the same shortcut adjustment settings as in Figure 3a. Namely, we experiment with combinations of sinusoidal encodings, learned encodings, the sum of sinusoidal and learned encodings (rows in the figure), and the choice of concatenating positional encodings with the amino acid embeddings or combining them by addition (columns in the figure).

While there are overall only small differences in performance between the positional encodings, we have conducted our studies with BASE-T and FINE-T models using the combination of sinusoidal and learned positional encodings, which are added to the amino acid embeddings (bottom right subplot) since this setting has an overall slight advantage in performance, especially when compared to the setting with sinusoidal encodings that are concatenated to the amino acid embeddings.

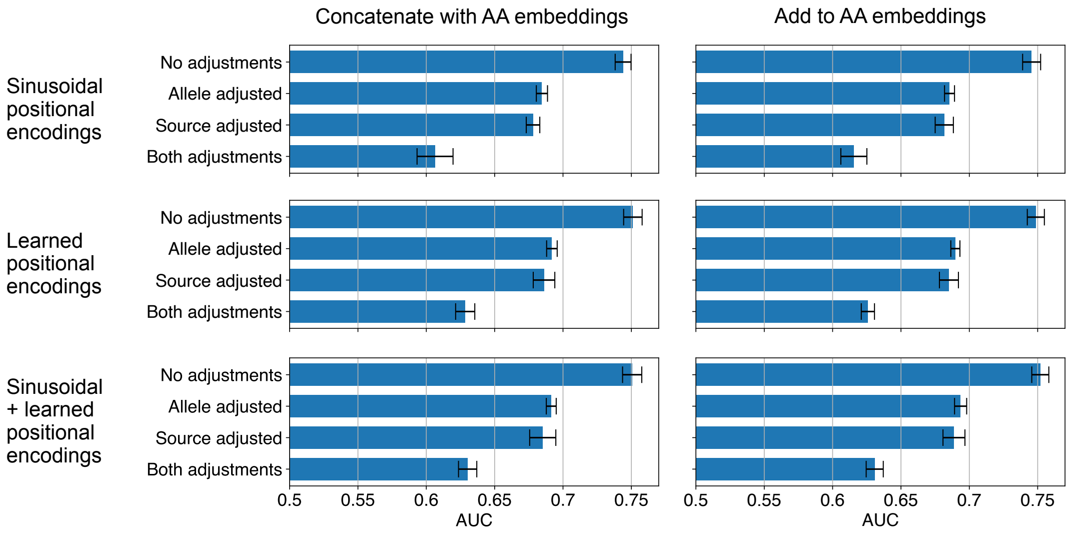

S 5 Selection of domain adaptation strength in ADA-T

The domain adaptation strength λ is the hyperparameter of ADA-T that determines how strongly the domain invariance should be encouraged by the model. This is why we select λ not only based on the predictive performance but also on the reduction of domain-based shortcut learning. To optimize the predictive performance, we aim to select a low value of λ, and to avoid shortcut learning, we aim to minimize the difference between the performance without adjustments and the shortcut adjusted performance. The results below show the performance of ADA-T applied to peptide source domain adaptation with $\lambda \in \{2, 4, \ldots, 26\}$. We select $\lambda = 10$ since it is the lowest value that leads to similar performance without adjustments and with source adjustment, as measured by overlapping error bars.

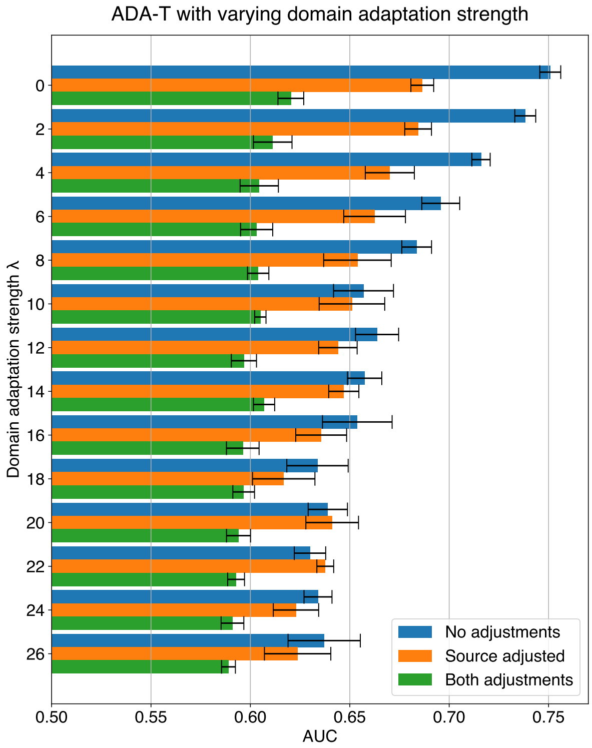

S 6 Shortcut learning influence on prediction scores

Similar to Figure 3b and Figure 3f, we show the distributions of prediction scores separated by the ground truth label for both the BASE-T model and the ADA-T model. In contrast to Figure 3b/3f, each peptide source is shown as a separate row of histograms.

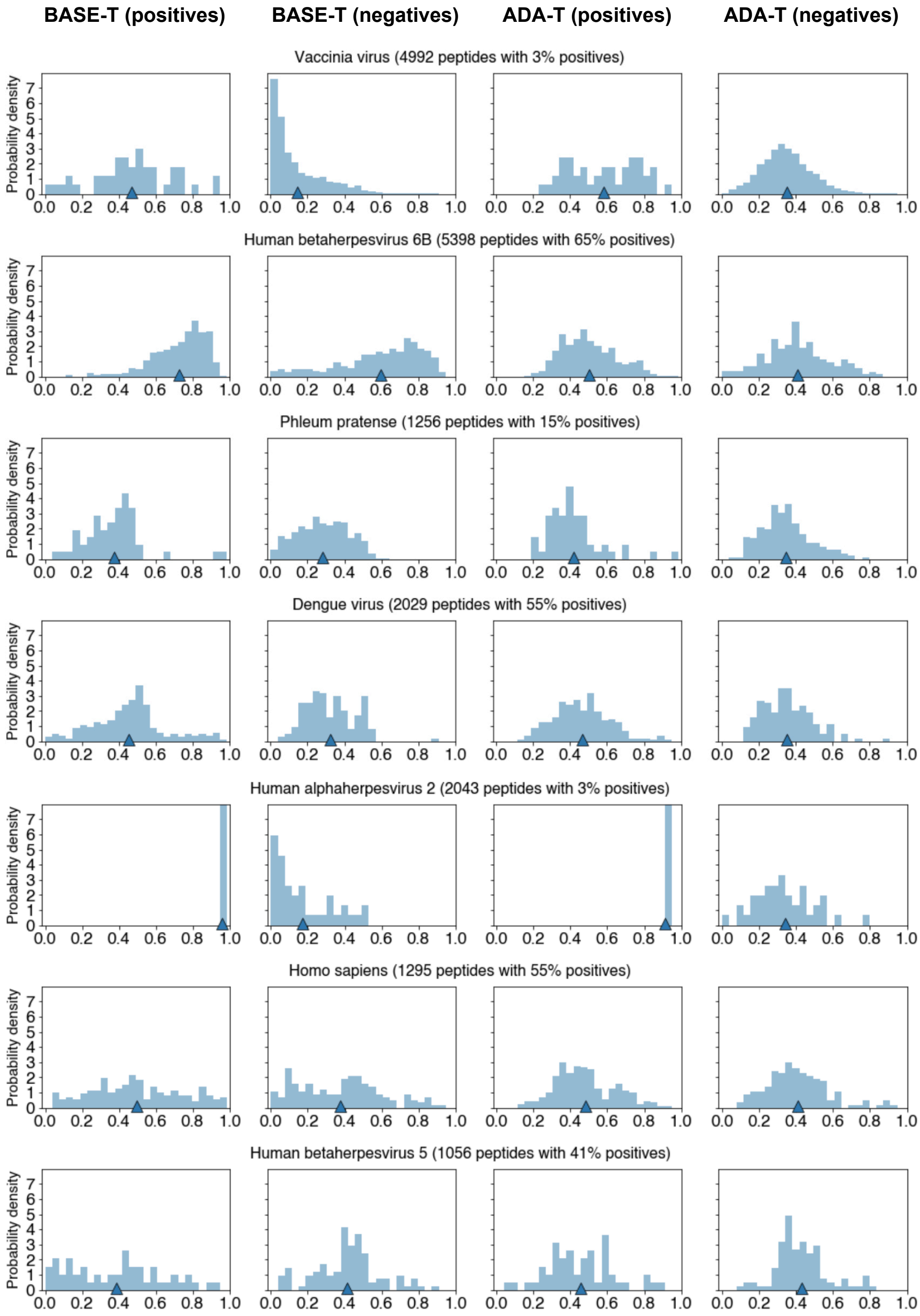

S 7 Similar sequences within and between peptide sources

To investigate possible causes for negative transfer between peptide sources, we analyze how similar sequences are to each other within a peptide source, and when compared to peptides from another source. As a sequence distance metric we use length-normalized Levenshtein distances. We plot the distributions of within-source and between-source Levenshtein distances for the ten most frequent peptide sources in the histograms below. For each peptide of a peptide source, we plot the Levenshtein distance to the closest peptide of the same source (blue histogram bars) and to the closest peptide coming from one of the other peptide sources (orange histogram bars), always requiring matching T-cell response labels. Additionally, we annotate the 10th percentiles of Levenshtein distances for these two settings.

Considering the ten most frequent peptide sources, the plot below shows that for eight of them, the 10th percentile is smaller for within-source distances than for between-source distances. Having very similar peptides with matching labels within a source suggests an easier prediction task. In contrast, there are fewer similar peptides with matching labels between sources, which might lead to negative transfer between the sources.

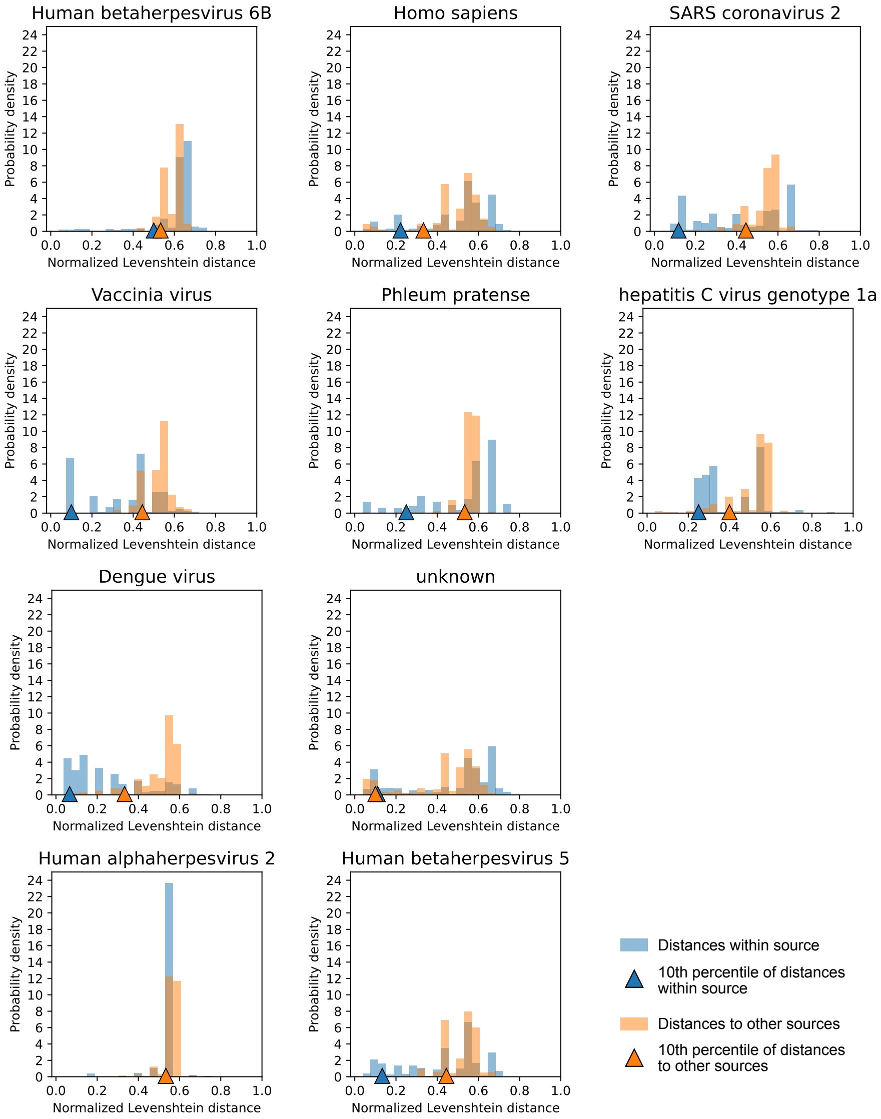

S 8 Negative transfer between domains

As described in the main paper, we compare BASE-T, being trained on all peptide sources and MHC alleles (multi-domain setting), with a combination of BASE-T models that eliminate transfer between sources and alleles respectively.

The following plot shows the validation performance of these three settings. The observation that per-source models and to a smaller degree per-allele models lead to more accurate predictions than the multi-domain model implies that the multi-domain model suffers from negative transfer between peptide sources and to a smaller degree between MHC alleles.

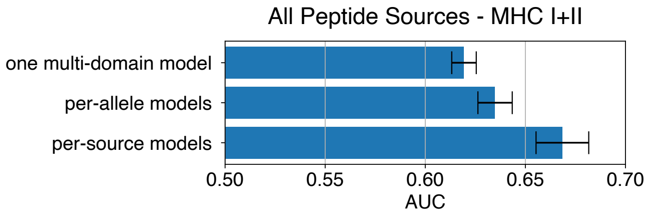

S 9 Transfer to unseen peptide sources

We compare the BASE-T model trained on all peptide sources with several versions of BASE-T trained on all sources except the one source that we use for the evaluation. The peptide sources in each plot are sorted according to the number of samples (largest on top).

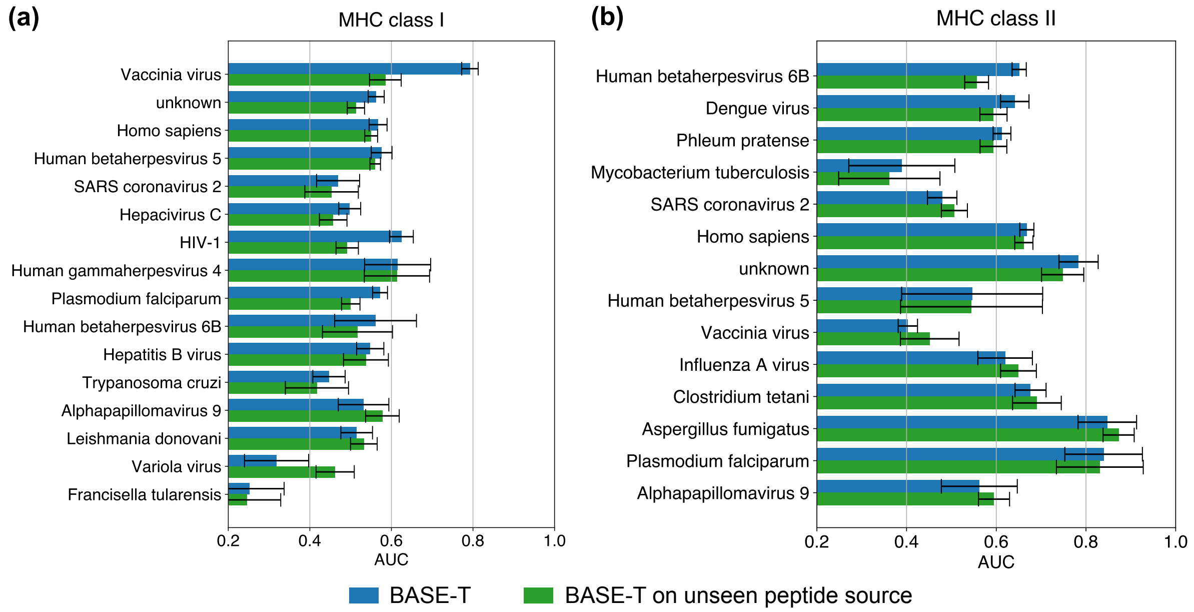

S 10 Study of FINE-T on more peptide sources

This study extends the analysis of FINE-T to a larger set of peptide sources, which also includes infrequent sources. The peptide sources in plots (a) and (b) are sorted according to the number of peptides per source (largest on top). The respective numbers of peptides per source are shown in part (c) and (d).

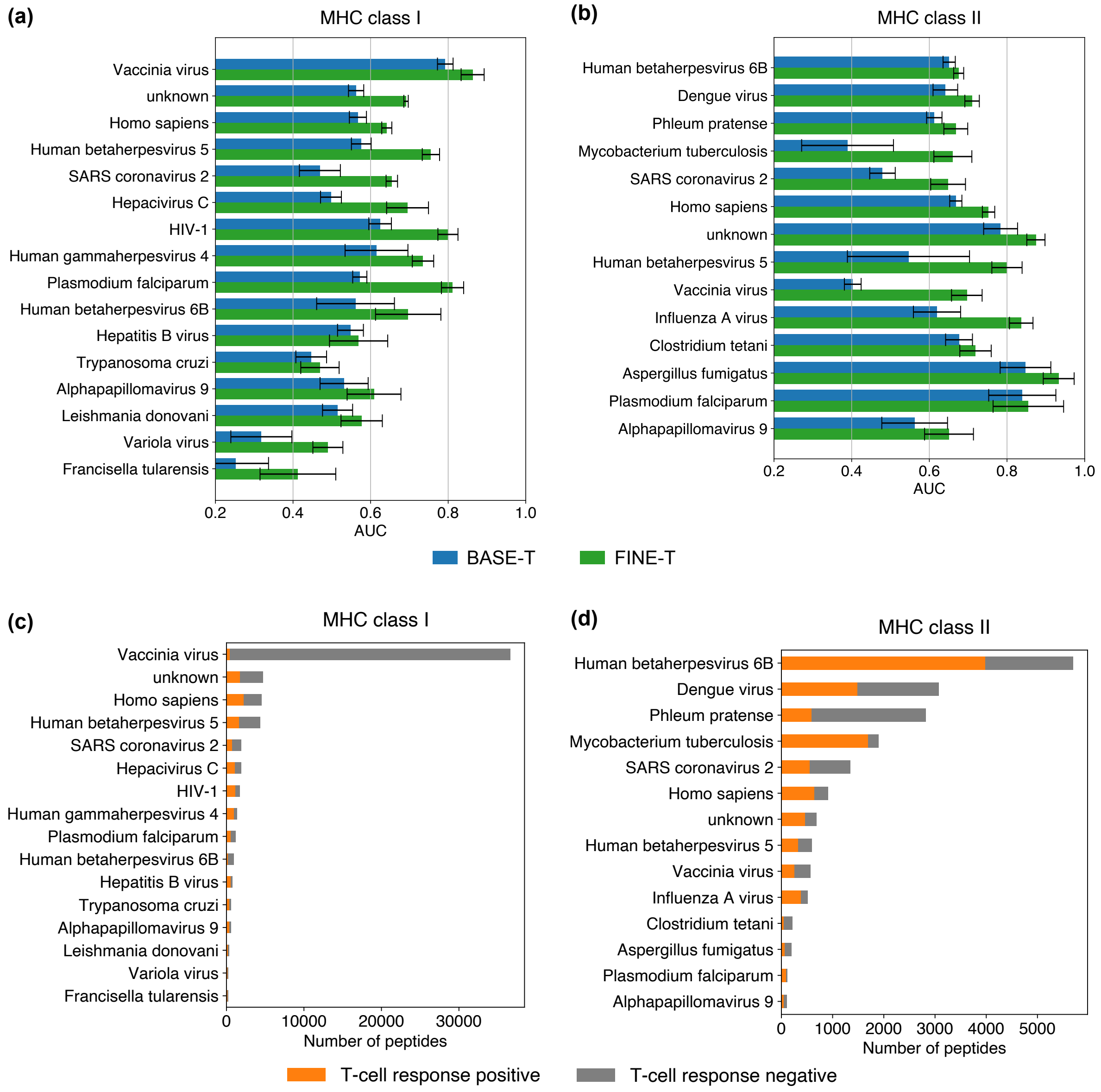

S 11 Ablation study for FINE-T

To investigate the contribution of fine-tuning and the benefit of mitigating negative transfer, we show the validation performance of transformer models in different transfer learning settings for several peptide sources.

Results for MHC I are shown in **(a)** and for MHC II in **(b)**. For each MHC class, the five most frequent peptide sources are selected. Peptide sources with only positives or only negatives in one of the data partitions are excluded.

This analysis shows that both mitigating negative transfer (multi-domain vs. per-source) and fine-tuning (FINE-T vs. per-source) consistently improve the predictions.

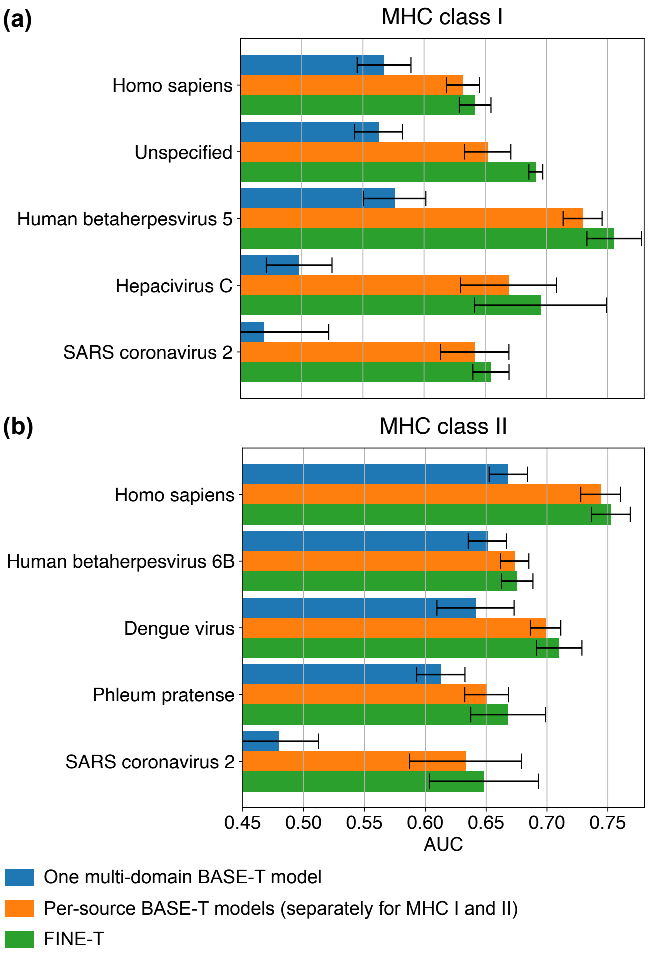

S 12 Permutation experiment with FINE-T

We randomly permute the labels of training and test data sets 200 times to obtain the performance distribution of the FINE-T model when only random patterns are picked up. Results for MHC I are shown in **(a)** and for MHC II in **(b)** in the following figure.

We apply the permutation always within the training data set and within the test data set and apply the same training and evaluation procedure as for obtaining the test performance in Figure 5 (grey bars).

The red arrow indicates the performance of the FINE-T model when trained and evaluated on the data sets without permutation. Consequently the red arrows show the same performance as the grey bars for FINE-T in Figure 5. Since the red arrows lie outside of the performance distribution of the permutation experiment, the performance of FINE-T cannot be explained by learning random patterns.

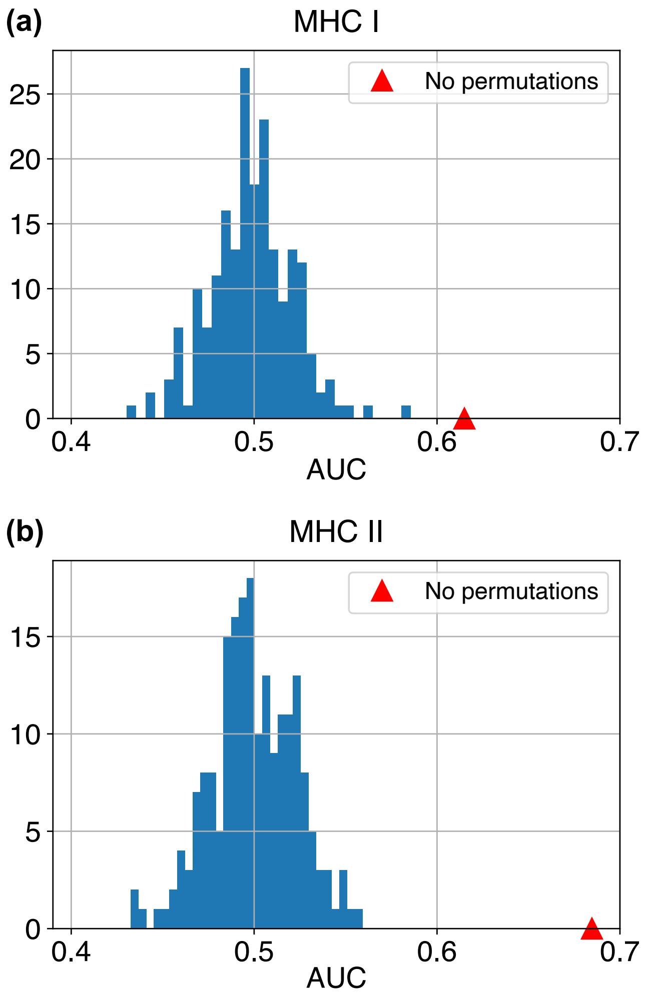

References

Kingma, D. P. and Ba, J. (2015). Adam: A method for stochastic optimization. In *ICLR (Poster)*.

Paul, S. *et al.* (2015). Development and validation of a broad scheme for prediction of HLA class II restricted T cell epitopes. *Journal of immunological methods*, **422**, 28–34.
